# Garment Counting in a Textile Warehouse by Means of a Laser Imaging System

**DOI:** 10.3390/s130505630

**Published:** 2013-04-29

**Authors:** Alejandro Santos Martínez-Sala, Juan Carlos Sánchez-Aartnoutse, Esteban Egea-López

**Affiliations:** Department of Information and Communication Technologies, Universidad Politécnica de Cartagena, Campus Muralla del Mar, Cartagena E-30202, Spain; E-Mails: juanc.sanchez@upct.es (J.C.S.-A.); esteban.egea@upct.es (E.E.-L.)

**Keywords:** textile warehouse, garment counter, laser imaging, phototransistor, image processing

## Abstract

Textile logistic warehouses are highly automated mechanized places where control points are needed to count and validate the number of garments in each batch. This paper proposes and describes a low cost and small size automated system designed to count the number of garments by processing an image of the corresponding hanger hooks generated using an array of phototransistors sensors and a linear laser beam. The generated image is processed using computer vision techniques to infer the number of garment units. The system has been tested on two logistic warehouses with a mean error in the estimated number of hangers of 0.13%.

## 1. Introduction

**Figure 1.**
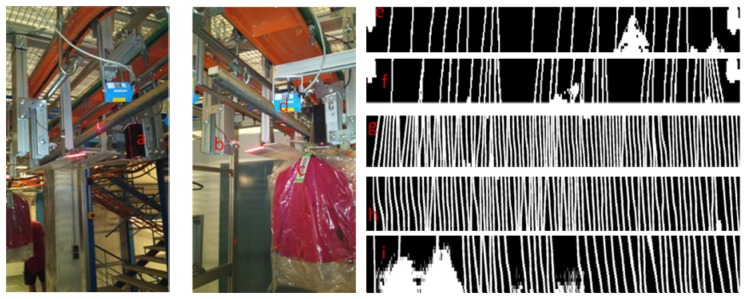
Picture of a garment counter device based on laser imaging in a textile warehouse; (**a**) sensor array and image acquisition electronics, (**b**) laser lineal emitter, (**c**) bar code label for internal traceability of a batch of garments in hangers, (**d**) bar code scanner, (**e**,**f**) examples of generated images of hanger hooks to estimate the actual number of garment units in a trolley, (**g**,**h**,**i**) examples of generated images in continuous chain mode. In (i) noise can be seen due to plastics.

The purpose of this paper is to present a novel automatic system designed to identify and count the number of garments hanged in a batch which is moved, transported and managed within a textile warehouse (Figure 1). The system consists of a linear beam laser emitter which hits on a linear array of sensors based on phototransistors that generate an image of the visible part of the clothes hangers. This image is processed to compute the number of cloth units of a batch with low error. In this section, the operation of a logistic textile warehouse is described and its needs discussed. In Section 2, we propose a novel laser system for garment counting, describe its operation and components and discuss the main design decisions. In Section 3, we show preliminary results of the use of the system in a fully-operated industrial environment. Finally, in Section 4 conclusions are future lines are summarized.

### 1.1. Textile Warehouse Operation and Needs

The supply chain of large textile companies consists of: (1) a network of garment producers, (2) logistic intermediate textile company warehouses and (3) the final network retail stores. Textile logistic warehouses incorporate advanced computer systems and automated complex mechanical systems to: (1) manage hundreds of thousands of units of clothing a day; (2) to receive orders from a manufacturer, (3) to classify and (4) register the clothes in the computer system, (5) to store and (6) prepare orders for stores. In large textile warehouses, clothing units in stock may number around the million units. Shipping and storage of clothing can be done in cardboard boxes or hanged on hangers. Therefore, the mechanical systems of logistics textile warehouses are designed to manage clothing in boxes or hanged in hangers. Regarding the clothing hanging on hangers, nowadays there are two major mechanical systems for internal management of batches of clothes: trolleys system and continuous chain system.

**Figure 2.**
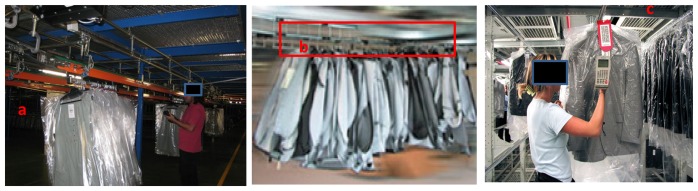
(**a**) Trolleys system. Operator counting and validating with a PDA the number of clothing units. (**b**) Continuous chain system. (**c**) Storing area of a batch in continuous chain system.

A trolley is U-shaped and comprises two vertical and one horizontal bar, which supports the clothes hangers (see Figure 2(a)). The vertical bars are coupled to a continuously moving chain system which pushes and moves the trolley throughout the warehouse. The continuous chain system is similar in concept, except that the horizontal bars of its links are used to hang the clothes hangers. An important difference to take into consideration is that in a trolley, the horizontal bar measures approximately one meter, and so the number of garments it can handle is limited. Whereas the continuous chain system consists of batches with a varying number of garments, and thus, the linear distance is up to 5 meters.

Clothes received from a manufacturer come in a truck hanged on hangers and they are manually unloaded. Then the garments are placed in the warehouse transport system (trolley mode or continuous chain mode) generating small batches of clothes of the same type (model, color, size) of variable size.

Operators manually count the number of units on each batch. For the sake of internal traceability a barcode is associated to each batch, encoding the type of clothes and the number of units. All these data is then recorded and stored in the computer system. This process is prone to errors: as with any manual process, human errors happen during the counting and registering process of the actual number of units in a batch [1]. In addition, for efficiency reasons the time spent counting and checking on the number of units of a batch must be as small as possible.

There is an extensive rail system (in the order of kilometers) that carries and manages the hanging clothes within the warehouse, even among several levels or floors, for both operation modes (trolley mode and continuous chain mode). The number of units in a batch may change because during the internal transport of batches, there is a probability that clothes fall.

Therefore, despite of the demanding operational processes, the operator's training and qualification, the high degree of automation of a textile warehouse and the sophisticated computer systems used, small stock variations may occur due to various faults. Considering that in a textile warehouse, within a short period of time, the flow of units can be in the hundreds of thousands, these fault-induced variations in the stock may quickly become relevant with the passage of time.

To computationally validate the actual number of units of a batch is an important logistical information task. Therefore, and depending on the structure and organization of a textile warehouse, check points are set up in strategic areas where an operator: (1) reads the internal traceability batch barcode with a PDA; (2) manually counts the number of units of clothing and confirms or modifies the number of units in the PDA to update the management information system. Obviously, this is a tedious job for operators, which is prone to human errors in the counting process [1].

In summary, in a textile warehouse, with a high flow of hanging clothes on a hanger, there is a clear need for an automated system which identifies batches of clothing, counts the actual number of units and updates the data in the computerized warehouse management system. Such a system is expected to increase productivity and reduce logistics time.

### 1.2. How to Count Clothing Units in an Industrial System?

The remarkable variety of models, different sizes and colors as well as the season variations (summer or winter) makes difficult to find a uniform and steady pattern for automated identification and counting of units of clothing. We start from the hypothesis is that the hook of the hanger can be a stable and suitable pattern for counting because there is usually a one-to-one correspondence between a hanger hook and a garment. This hypothesis is valid for both operation modes: trolley and continuous chain mode. Additionally we consider that the shape, type and thickness of the hangers used in a batch is always the same. Therefore, the technical challenge is how to design an efficient system to count the number of hooks of hangers to infer the number of garments. In addition such a system should be able to detect the presence of a batch of clothes and read their internal traceability barcodes.

Moreover, the following operational particularities have to be taken into account in the design of the system. First, the main difference between a batch of clothes in trolley mode or continuous chain mode is the maximum linear distance between the first and last hanger of each batch: less than one meter in trolley mode and up to 5 meters in continuous chain mode. Second, the number of hangers of one batch is variable and depends on several factors:

- Type of season clothes: In the spring and summer campaign, garments are thin and lightweight so the batch can be densely loaded with hangers. In the fall and winter season, the clothes are of greater volume, e.g., coats, so the average number of garments is smaller.
- Quantity of goods available: Loading and batching depends on the number of units of a type of clothing that is available, so there can be batches fully loaded, medium or low loaded.

It should be also noted that when the hanger wears clothes and it is hung on a bar, the visible metallic portion of hook is approximately 50 mm. Ideally, the visible part of the hanger hook should provide a stable pattern. Unfortunately, during the transportation of a batch the garments are moving and swinging, therefore they collide with each other, overlap and cross each other so that they are not perfectly aligned and spaced. Finally, the hooks of the hangers may carry cellophane wrapping and plastic protective apparel may protrude, partially hiding the hangers.

### 1.3. Existing Technical Solutions

As far us we know, there is a lack of scientific literature related to optical or mechanical garment counters. Furthermore, there are no general-purpose commercial devices designed for this task. Therefore, in the following paragraphs we describe related patents and similar systems for the sake of comparison.

Ideally, if the garments did not move, the hangers would stay perfectly aligned and separated and, then, a single mechanical detection sensor could be used to count the number of units. These approaches have been used for the last 40 years but have not evolved much since the issuance of patents such as [2], where a specially shaped hook is required. As a main drawback, the system requires that the hooks be perfectly aligned with the sensor. A later approach was presented in patent [3]. It main drawback is that it requires special hangers with cutouts in a portion of the hook; therefore, standard hangers cannot be used. Moreover, the system does not work properly if the hanger is not perfectly aligned. Another possible solution is to associate a passive RFID tag to each unit of clothing and place RFID portals in the main checkpoints [4–6]. Its main drawbacks are: (1) this is an expensive solution under research, and (2) clothing manufactures are not willing or ready to change their current barcode labeling systems to RFID [7].

**Figure 3.**
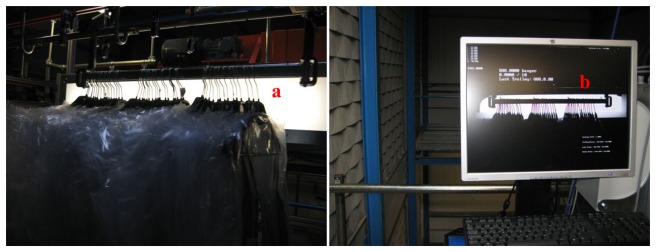
Garment counter based on a vision system: (**a**) Trolley and additional illumination to improve the image acquisition. (**b**) Trolley's picture from a camera.

On the other hand, to the best of our knowledge, counting units of clothing based exclusively on image processing have not been proposed in the bibliography. There are some approaches to test and analyze the quality of the clothes using image processing such as [8], but these approaches are not suitable for counting garments because their purpose is completely different: automate recognition of garment quality. For that purpose, a complete picture of the garment is needed; therefore each garment should be extracted from the trolley and inserted again when the process is done. In other fields of application, there are different approaches to use image processing to count items, such as fruit classification systems that can be used to count [9]. These solutions can not be applied to the problem proposed in this paper because (1) the area of interest is quite long and (2) it is not fixed. That is, the main problem of these solutions is the potentially large extension of the area of interest. They can be valid for the trolley mode because its limited and known size makes it feasible to use a camera to capture a picture of the area of interest (see Figure 3). However, vision systems are not appropriate in continuous chain mode because the linear length of a batch is variable and up to several meters. It requires a complex and expensive camera system deployed on a wide area for image processing. Furthermore, in textile warehouses the available space is usually highly optimized and thus limited, which makes even difficult to install a vision system. In short, systems based exclusively on artificial vision are only appropriate for trolley mode and their price is too high to be amortized in a reasonable time.

Our solution, on the other hand, uses image processing to identify and count the number of hangers, but, in order to limit the size and complexity of the required system, the image is generated by a laser imaging device, which is a novel contribution.

In the following section we propose and describe the architecture of our system for identification and counting garment units based on the processing of an image generated by a laser beam. The main benefits of the proposed system are its low cost, multimode operation (trolley and continuous chain mode), and reduced size.

## 2. Garment Counter Based on a Novel Laser Imaging Approach

In the following sections the garment counter is described and discussed in a top-down approach.

### 2.1. Architecture of the System

**Figure 4.**
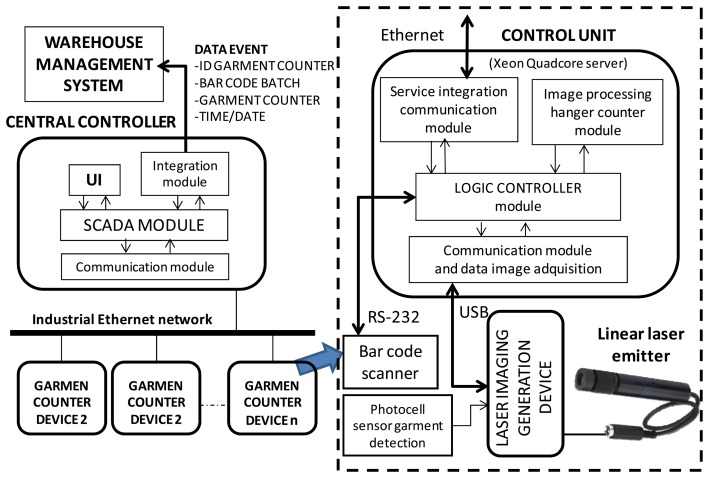
Central server connected to the network of garment counter devices. Integration with the warehouse management system. Functional blocks of the GCD.

Our system is based on the deployment of novel Garment Counter Devices (GCD) in the main control/check points of the textile warehouse. These devices communicate with a Central Controller (CC) via a standard Ethernet network (see Figure 4). The Central Controller implements a Supervisory Control and Data Adquistion System (SCADA) [10] module dedicated to monitor and manage on-line the Counter Devices. When an alarm arises, or when a technical problem occurs, a visual message pops-up at the User Interface (UI) warning of such an event to a maintenance operator. Additionally, when a batch of garments pass through a control point, a message is delivered to the Central Controller, which immediately adapts the message format to the communication and integration requirements of the warehouse management system. In order to guarantee traceability, the messages forwarded to the warehouse management system must contain: (1) the GCD identification number (*i.e.*, check point identification), (2) the batch barcode (which codes type of clothes, *etc.*), (3) the number of garments counted by this device, and (4) the data and time of that event.

### 2.2. Garment Counter Device Based on Laser Imaging

**Figure 5.**
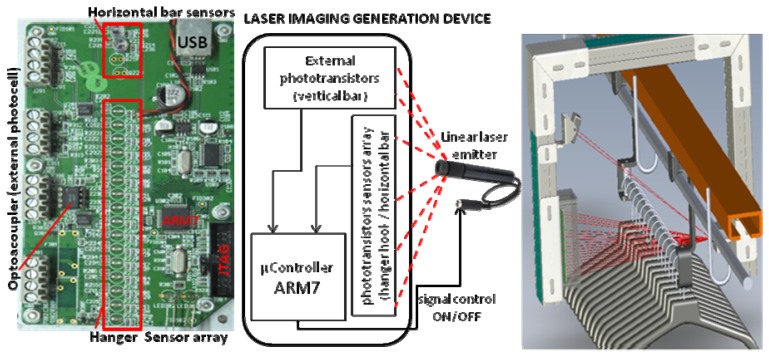
Laser imaging Generation Device.

**Figure 6.**
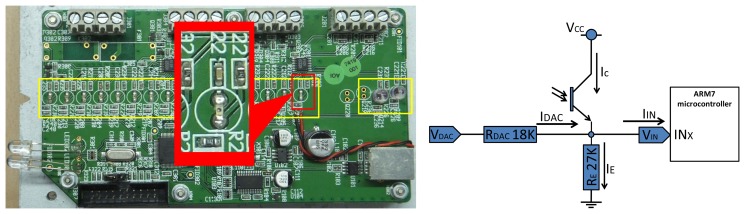
(**a**) Electronic board with the phototransistor sensors array. (**b**) Detailed picture of the phototransistor circuit. (**c**) Schematic of the phototransistor circuit.

**Figure 7.**
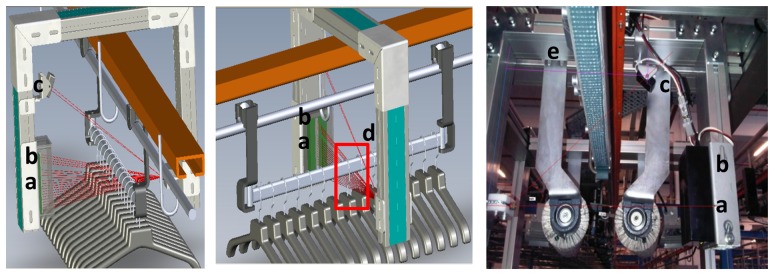
Physical representation of the laser signal exciting the sensors or intersecting with the garments’ hangers. (**a**) hangers’ sensor array, (**b**) Horizontal bar sensors, (**c**) Vertical bar sensors, (**d**) target intersecting zone between the laser beam and hanger hooks, (**e**) Real system installed.

Figure 5 shows the main blocks and elements of a GCD from three perspectives: conceptual blocks, physical implementation on the PCB and physical location and interaction in a control point of the warehouse. Laser imaging generation device block consists of an ARM7 microcontroller, which mainly samples the information captured by a set of phototransistors (grouped by functionality in three groups of 16, two and two sensors. These phototransistors are lighted by a laser emitter [11], which is also controlled by the microcontroller. A physical representation of the sensors and the laser emitter is also depicted in Figures 1, 6 and 7.

The ARM7 provides a serial-USB interface to communicate with the Control Unit (CU). Communications are implemented by a master-slave protocol and used to set the ARM7 operation mode (configuration, calibration, trolley or continuous chain mode).

Figure 6 shows the physical implementation of the circuit board. In the picture, all the phototransistors [12,13] have been marked by yellow boxes: an array of 16 sensors and another two grouped together. There are two external sensors that are not depicted in Figure 6, but can be seen in Figure 7. All the phototransistors sensors are excited when they receive energy from a laser beam (in Figure 7 it is shown the reception of trays at the sensors). Figure 6(c) depicts an electronic scheme of a phototransistor sensor. Notice that each phototransistor's emitter (referenced by Vin) is connected to a digital input of ARM7 microcontroller, which periodically samples the value of such inputs. The microcontroller assumes as a logical “1” at the related digital input when Vin offers values above 2 V. On the other hand, if Vin is below 0.8 V a logical “0” is assumed. In Section 2.2.1 we will explain in depth the behavior of this scheme.

This design decision of using the digital inputs of the ARM7 is due to the need of simultaneous sampling of all the sensors (16+2+2) in real time. The main advantage of this design is that it can read all the digital inputs in just one instruction cycle.

The alternative is to use an external ADC module, but typical ADC sampling and response times do not meet our time constraints. In addition, in this solution, the data should be transmitted to the ARM7 via an I2C or an SPI bus, and stored in a register, which also takes more time. Therefore, the delay (and the latency) will be higher, which directly affects to the real-time operation of the sampling and processing program, which may lead to an overflow or data loss. According to their purpose and the information provided by them, phototransistors used in the device can be classified into three types:

- Hanger sampling sensors: 16 sensors which are responsible of generating an image of the visible area of the hanger's hook (left yellow box in Figure 6(a)).
- Horizontal bar sampling sensors: two redundant sensors dedicated to detect the horizontal bar on which hangers are hanged (right yellow box in Figure 6(a)).
- Vertical bar sampling sensors: these sensors are external to the main electronic. Their aim is to detect the vertical bar of a trolley, or the vertical bar of a link in a continuous chain mode. See detail in Figure 7.

When the beginning of a batch of garments is detected, in either trolley mode or continuous chain mode, digital inputs of the hangers sampling sensors are sampled creating a vector with the digital word <S0,S1,S2,S3,S4…S15> with the instantaneous state of each sensor. Note that sensor S0 is the upper hanger's sensor and S15 is the lowest one. Every sampling period, the ARM7 sends a message with this array of data (encoded by 2 bytes) to the Control Unit, which stores the data in a reception buffer. The Control Unit will reconstruct the image associated to the hangers’ hooks.

If we assume that a hanger is statically positioned and aligned between the array of sensors and the laser emitter, the laser beam must hit on a small portion of the hanger hook, concretely of 50 mm. This area has been chosen because it provides the higher probability of offering a clear vision, that is, that the hook is more visible and there are no obstacles (mainly clothes).

The device uses 16 uniformly distributed sensors to digitalize a piece of hook of 50 mm length by means of 16 real points spaced 3.125 mm. These 16 points provide enough information to characterize and create an image of the area under study. In addition, the data vector can be encoded in only 2 bytes, therefore the implementation of data transmission protocol between the microcontroller and the Control Unit is simplified.

When the laser beam is completely eclipsed by a hanger's hook, any hanger sampling sensor receives energy from the laser beam, and therefore the corresponding data vector encodes a logical “0” in every sensor. This information is represented by white pixels in an image.

The array of sensors have been distributed uniformly on an straight line of 100 mm on the PCB; and this PCB has been placed 200 mm far away from the horizontal bar (where the hangers are supported) and 400 mm far away from the laser emitter. This distance of 400 mm has been chosen as a trade-off between two extreme design requirements: (1) the device has to avoid crashes with clothing, and (2) the system has to be easy to install in any place in the warehouse (that is, as small as possible).

As mentioned above, the main task of the microcontroller is to sample the sensor data and send that data to the UC. Nevertheless, the microcontroller has to perform other functions according to the operation mode. The ARM7 microcontroller can be used in the following modes:

- Configuration Mode: the Control Unit sends a command to the ARM7 to enter this mode and set parameters of the phototransistors sensors such as sample rate and calibration.
- Calibration mode: the Control Unit sends a command to the ARM7 asking to send all digital inputs from existing sensors: vertical bar sensors, horizontal bar sensors and hangers sampling sensors. Calibration operation is performed with this data.
- Trolley Mode: Control Unit sends a command to the ARM7 to run a state machine that detects the beginning of a trolley, generates the corresponding image of the hangers and detects the end of the trolley.
- The state machine of the microcontroller processes the instantaneous values of the digital inputs of the horizontal bar sensors and vertical bar sensors to decide whether it is the beginning or the end of a trolley. The values of these digital inputs are not sent to the Control Unit.
- Continuous chain mode: Control Unit sends a command to the ARM7 to run a state machine that detects the beginning of a batch of hangers of variable length, generates the corresponding image of the hangers and detects the end of the hangers’ batch.

The communications module and image acquisition block of the Control Unit (see Figure 5) stores in the reception buffer the data vectors sent by the ARM7. These data are used to create a 16×N image matrix where:

- The i-th row corresponds to the data sampled by the digital input of the i-th sensor. Each hanger sampling sensor is a pixel of a black and white image.
- Each column corresponds to a vector of data sampled at a given time.
- The matrix has N columns corresponding to the N sampling instants; the first instant takes place when the hangers’ batch detection event is generated, whereas the N instant happens when the end of batch event is generated. For image processing requirements, the logic of the data stored in the array is inverted: a pixel “0” is black and a “1” is white.

Let us note that in a trolley, the number of samples is constant because all of them have the same length, therefore the pattern used to detect the beginning and end of a trolley remains the same. On the other hand, in the continuous chain mode the number of samples of a batch, *i.e.*, the number of columns of the image matrix, is variable due to the fact that the linear distance from the first batch's hanger to the last one is not constant. Finally, after the points of the columns of the binary data matrix are interpolated, a black and white image is generated. This image is equivalent to a real image obtained with a camera.

#### 2.2.1. Calibration Process of the Digital Inputs

As explained in previous section, depending on the irradiance of the laser beam on the surface of the phototransistor, the Vin signal can be:

- Vin < 0.8 V, which means a logical “0” in the digital input. When a phototransistor is in cut-off state a logical “0” is showed.
- 0.8 < Vin < 2 V, represents an uncertainty in the digital input, it is not possible to assign a logical “0” or “1”, therefore it is a undesirable situation.
- Vin > 2 V, which means a logical “1” unambiguously. In order to ensure that Vin is logical “1”, the phototransistor shall work in saturation state or active state but offering a Vin high enough. Additional voltage supplied by DAC helps to achieve that voltage.

When a hanger passes at constant speed and it is sampled at a constant frequency, the amount of energy that a phototransistor sensor receives can changes amongst these cases:

- There is no obstacle between the sensor and the laser beam. Therefore the sensor receives enough energy on its surface and Vin generates a logical “1” (equivalent to a black pixel).
- The hanger's hook starts to eclipse a little bit the sensor's surface. Therefore the energy received by the laser beam decreases.
- The hanger completely eclipses the sensor's surface. It does not receive any energy from the laser beam, thus Vin is a logical “0” (equivalent to a white pixel).

It would be desirable that all digital inputs read the the same logical value “1” or “0” under the same eclipsing circumstances. Unfortunately, given the same obstacle, phototransistor sensors do not receive the same energy and there are variations in signal Vin for several factors:

- The power density of the laser beam is not perfectly linear and follows a Gaussian distribution; the sides of the beam are less powerful [14].
- The sensors in the central area receive perpendicularly the rays of the laser beam. On the other side, sensors at the extremes receive more inclined rays which reduces in some degree the received power.
- As in every component, there are manufacturing tolerances in the phototransistors. There can be differences in the relationship between the current in the collector for the same received energy.

The calibration process of the digital inputs associated to each sensor aims to normalize the values of digital inputs when the sensors are eclipsed by the same obstacle. This calibration is done by modifying the DAC reference voltage provided individually for each phototransistor.

The microcontroller commands the DAC through an SPI bus. The required V_DAC_ is set by means of a byte that codes the corresponding output voltage supplied to a phototransistor. According to the byte value (from 00h to FFh), 256 levels of tension between 0 V and Vcc can be encoded.

In the calibration process, the Control Unit sends a command to the microcontroller to enter in the calibration mode. Then, the microcontroller sends raw data vectors from all digital inputs associated with the sensors at the sampling frequency. The calibration process is iterative and heuristic and it can be split in the following steps:

- A perfect pattern of a hanger (a straight wire of constant thickness) is passed completely parallel though the device, at the constant chain speed to the array of sensors.
- The Control Unit receives the raw data. Then the generated image is checked against the pattern. There will be digital inputs with differences in the generated white pixels. The DAC registers of sensors are heuristically changed. The pattern is passed back and the resulting image is again analyzed.
- The process is repeated until all the digital inputs generate the same amount of white pixels under a tolerance of 2–4 pixels.
- The values are stored in the DAC registers, and, in configuration mode, the values are permanently stored in the microcontroller.

From the point of view of the phototransistor behavior, the theoretical estimation of the maximum and minimum V_DAC_ values can be calculated as follows: when a phototransistor receives the minimal amount of energy to activate it, the emitter current is the sum of the collector current plus the current from the V_DAC_ (Equation (1)).

(1) $$\begin{array}{c} I_{\text{DAC}}+I_{C}=I_{E}\\ \left(I_{in}=0,Z_{in\_\text{ARM}7}\to \infty \right) \end{array}$$

Replacing values and reordering, Vin leads to:

(2) $$\begin{array}{cc} \frac{V_{in}-V_{\text{DAC}}}{R_{\text{DAC}}}+I_{C}\left(E\varepsilon \right)=\frac{V_{\text{DAC}}}{R_{E}} & \left(E\varepsilon \left(\frac{mW}{cm^{2}}\right),\text{irradiance}\right) \end{array}$$

In those situations where a logical “1” is expected at the microcontroller input, but Vin provides a value lower than 2 V; the value of V_DAC_ must increase in order to satisfy the inequation. Rewriting the above equation leads to:

(3) $$V_{\text{DAC}}=V_{in}\cdot \left(\frac{R_{E}-R_{\text{DAC}}}{R_{E}}\right)-I_{C}\left(E\varepsilon \right)\cdot R_{\text{DAC}}$$

On the other hand, V_DAC_ can not be as high as we would like to. It must be taken into account that in the presence of an object which completely eclipses the sensor, the voltage Vin have to be less than 0.8 V (0 if V_DAC_ = 0):

(4) $$V_{in}=V_{\text{DAC}}\cdot \frac{R_{E}}{R_{E}+R_{\text{DAC}}}$$

Now, rewriting the expression 1 and substituting the values, V_DAC_ can not exceed a value of 1.33 V:

(5) $$V_{\text{DAC}\_\max}=V_{in}\cdot \left(\frac{R_{E}-R_{\text{DAC}}}{R_{E}}\right)=0.8\cdot \left(\frac{27K-18K}{27K}\right)=1.33V$$

#### 2.2.2. Linear Laser Emitter

The emitter uses a continuous laser with a wavelength of 650 nm and an output power of 50 mW. The power voltage of the emitter is 5 V and it is turned on and off by a control signal generated by the ARM7 microcontroller. The emitter is focusable and its integrated optic generates a linear beam with a fan angle of 45°. The projected beam width is 200 mm, which mean that the sampled target portion of a hanger is less than one millimeter. The 650 nm wavelength has been used because it is a very common wavelength with a great variety of commercial modules with low prices. In addition, the laser beam is visible and it easies the mechanical adjustment and perfect alignment with the sensor's array.

#### 2.2.3. Industrial Photocell Sensor for Garment Arriving Detection

Textile warehouse activity is not uniform: there are peaks of great activity but also long periods of time without an activity of hanger batches. In order to increase the lifetime of the laser emitter, it's convenient to detect these inactivity periods where the laser emitter is not needed and can be switched off. For this purpose, an industrial photocell sensor has been installed. The photocell sensor is placed at clothing height and a few meters before the counting device. If there is an inactivity period long enough, the photocell sensor doesn’t change its signal and the microcontroller turns the laser emitter off. When batches are approaching to the counting device and passes through the sensor photocell, it generates a binary control signal to the ARM7 microcontroller which indicates that the laser emitter must be switched on.

### 2.3. Control Unit (CU)

The Control Unit uses a hardware server based on a Quadcore Xeon ×64 which has been designed for 24/7 lifetime operation. It runs Windows XP and .NET. The modules shown in Figure 5 have been programmed using C#, and following a Model-view-controller (MVC) architecture. The image processing module is implemented with Matlab compiled code [15,16], therefore, only the Matlab Runtime Compiler must be installed; the server does not need any other Matlab software.

The Logic Controller module runs the main control program and it is in charge of checking the whole system operation. In production mode, the Control Unit cyclically runs the following steps:

- The ARM7 periodically sends status information, even during inactivity periods. The Control Unit processes and forwards the status information to the Central Controller SCADA, which is in charge of monitoring services and remote maintenance.
- When the ARM7 detects the beginning of a hanger batch, the Control Unit receives a start message which means that it has to switch to “new hanger batch state”. Then, the Logic Controller module sends a SCAN command to the barcode scanner. Finally, the scanner returns the internal batch traceability barcode.
- The communications module and image acquisition block has a reception buffer where the data array is stored until the “end of batch” event is received, which means that the image matrix is ready to be generated.
- The Logic Controller module calls the image processing module, which is a Matlab compiled function, and passes as a parameter the image matrix. The image processing module returns as result the number of hangers calculated.
- The Logic Controller module creates a message with the identifier of the counter device, the bar code of the batch, the number of hangers and the date and time. This message is sent to the Central Controller. Finally, the Central Controller forwards the same new batch event to the warehouse information system.

#### 2.3.1. Barcode Scanner

The proposed system employs an industrial SICK barcode scanner. In the actual installation, two scanners are used (one on each side of the chain) to provide redundancy and to guarantee that all traceability barcodes of a batch are read. The communication interface with the Control Unit is via RS-232. In Figure 1(a) barcode label (Figure 1(c)) and the barcode scanner (Figure 1(d)) can be seen.

### 2.4. Image Processing/Hangers Counter module

**Figure 8.**
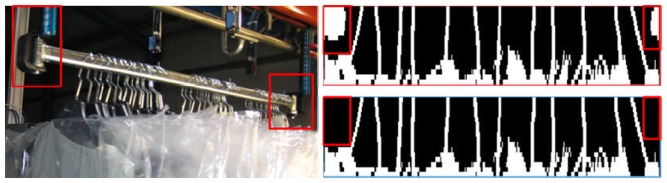
Picture of a trolley. Scanned image with edges. Scanned image without edges.

This module is programmed in Matlab because it provides a powerful image processing Toolbox to process image matrices. When the Logic Controller module calls the image processing module of Matlab, the input parameters are:

- Image binary matrix of 16×N elements.
- Calibration parameters of the hanger pattern: after the calibration process, the characteristic parameters of the hangers used at the facility are obtained and calculated. These parameters are used to identify the pattern of hangers, and to discern situations where the hangers are overlapping each other (represented in the image as a greater thickness hanger).
- Operation mode in the warehouse: the operating mode can be continuous chain or trolley. The operation mode has to be considered in order to apply a different treatment to the acquired image. For instance, a trolley has some parts, at the beginning and at the end, which are not hangers (see Figure 8). These parts have a well known pattern and it is necessary to remove them before the image of the hangers is processed. Once these edges are conveniently removed of the image of a trolley, the resulting image matrix is equal and equivalent to the image matrix obtained from the hangers of a batch in a continuous chain, the only difference is the total amount of samples.

**Figure 9.**
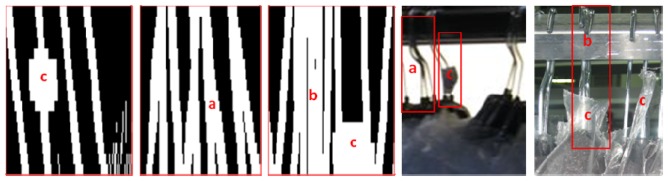
Cases and problems in real image and scanned image: (**a**) crossed hangers, (**b**) overlapped and aligned hangers, (**c**) obstacles in the image.

The purpose of the image processing is to isolate the visible area of the hanger hooks and to identify the characteristic pattern of a hanger [17]. In practice, there is a great variety of cases and problems to identify patterns of hangers (see Figure 9); though, they are identical in either trolley or continuous chain mode:

- Sets of crossed hangers: this situation is more likely when the clothes are thin and light. When the batch of hangers has suffered movements during its transportation, hangers are prone to cross each other. In these cases, is hard to count the hangers by visual inspection even for a human operator.
- Overlapped and aligned sets of hangers: during some process, the garments are compressed, such compression can lead to situations where two or more hangers are perfectly aligned, mounted over each other, giving the false perception of a thicker hook. In an extreme case even the gaps between hangers are vanished.
- Obstacles in the visible area of the hanger hook. Often, garments are wrapped with plastics. These labels and envelopes can hinder partly or completely the visible part of the hook. Other rare situations happen when hangers are reused and hook still have some cellophane from previous uses. The cellophane distorts the shape and thickness of a regular hook. A particular case occurs in clothing with high collars which reduces the visible part of the hanger to a very small and limited area. These situations are not a problem for a human inspector but it is a challenge for an automatic visual system.

#### 2.4.1. Calibration Process of the Hanger Pattern

Within a logistic warehouse, usually a type of hangers from a particular manufacturer is always used. However, the metal hangers’ hook does not follow a standard among manufacturers. Additionally, the counting system has to be installed in a control point of the warehouse where the chain speed is constant, but is not always exactly the same speed in every point. Therefore, the hanger's pattern has to be characterized for the specific type of hanger employed in the warehouse and for that specific control point. This process also is manual and iterative:

- In order to calibrate the device, a perfect pattern of hangers is passed through the device several times. The pattern is composed by a set of hangers that perfectly separated and without any kind of obstacles in the target portion of hook.
- That pattern is used to generate an image of the hooks without noise.
- The pattern is passed several times, enough for statistical estimation purposes.
- For each image, each hanger is isolated. Then the number of white pixels that are measured in the 16 points of the target segment are obtained. The number of white pixels of a sensor is proportional to the thickness of the hook at that point.
- A statistical processing is done from the data to characterize the measured thickness at each point measured by a sensor. For our purpose, the mean value is enough to characterize the thickness of the pattern used. This data is stored in the Control Unit. The image processing module will use this data as an input parameter in order to calculate the number of existing hangers in a batch.

Afterwards a set of real garments (not a perfect pattern of hanger) are passed to statistically calculate (1) the exceed threshold (to eliminate data from sensors with plastics) and (2) the size of overlapped garments.

#### 2.4.2. Heuristic Algorithm to Infer the Number of Garments

The algorithm for processing the image of the hangers obtained by the array of photodiodes has been developed heuristically. In this subsection their main steps are described.

**Figure 10.**
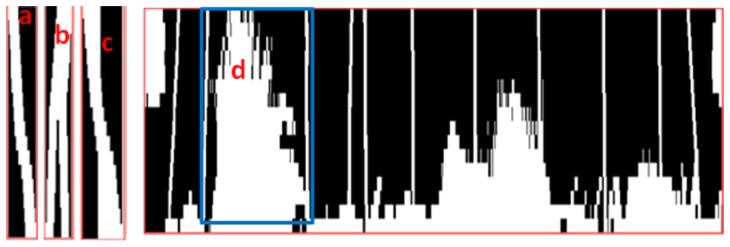
Examples of segmented sub-matrixes: (**a**) one hanger, (**b**) crossed hangers and (**c**) overlapped hangers. Main image matrix with noise (**d**): The obstacles are too high and are not possible to infer the actual number in the corresponding sub-matrix.

- Pre-processing of the main matrix (only for trolley system). In this step the well known pattern of the beginning and ending of the trolley is removed. This sub-module can be adapted and adjusted according to different trolley models (see Figure 8).
- Splitting the main matrix imaging: the main matrix is split into several sub-matrixes (which are easier and faster to operate) by means of looking for vector columns with all its elements marked as 0 (a “black”).
- Initial clustering to isolate likely groups of hangers. A neighborhood technique is applied to every sub-matrix, using the bwlabel function from the Matlab image toolbox. The purpose is to look for areas that are connected and surrounded by “0” (therefore we are looking for areas that could contain a hanger or a set of them). Then, a set of binary sub-matrix of 16 × M dimension and variable size are obtained, where the i-th row is the binary data measured by the i-th hanger sensor.
- For every binary sub-matrix and for every row (*i.e.*, sensor) the number of transitions from “0” to “1” (and “1” to “0”) is calculated. At the same time, the size of the segment between two consecutive transitions is calculated. When this segment's size exceeds a given threshold it is assumed that it is plastic and the data from this sensor should be eliminated from the sub-matrix.
- Finally, the number of transitions and their segment sizes per sensor is processed:
  ○ If all the sensors have two transitions and the mean size is under the overlapped threshold, it should be one hanger. See Figure 10(a).
  ○ If all the sensors have one transition and the mean size is above an overlapped threshold, it is very likely that there are two overlapped hangers and it is counted as two hangers. See Figure 10(c).
  ○ If there are sensors that have one transition and other ones have two transitions, it should be a crossed hangers’ case, then it is counted as two hangers. See Figure 10(b).
  ○ If there are too much obstacles in the image, it is impossible to compute the number of garments. See Figure 10(d).

## 3. Results

The system has been deployed and it is currently being tested in a Spanish company that has warehouses with both types of systems: a textile warehouse based on trolleys and another continuous chain-based one. In this section we provide the first results collected.

Prototypes of the system have been extensively tested in laboratory, obtaining good results. However, we cannot faithfully reproduce in the lab all the real problematic casuistry described in Section 3.3 (which is expected to occur in a real production environment). For that reason, some prototypes have been placed on selected check points of the warehouses.

The goal was to obtain a first proof-of-concept: that is, to confirm that the system is able to perform satisfactorily in a real non-stop production environment. For that reason, the counting system has been tested during the months of February and April in full production mode in order to evaluate the wide variety of types of clothing and problems patterns in the hangers.

The tests have been set as follows: the staff responsible for the warehouses systems have set two control points with fully-functional prototypes. The number of prototypes has been limited because the production cannot be altered in a significant way while the tests are performed: more prototypes require more staff and more delay due to the manual counting. Therefore, loaded trucks are handled as usually, but additional staff members have been placed to manually count the garments in the traditional way. This way, we double check the garment counting and obtain additional guarantees against human errors in the manual counting, which acts as the control samples. The double-checked manually obtained data have been compared with the data generated by the counting device in order to compare them statistically. Table 1 shows a sample of the data obtained in the continuous chain warehouse.

The totals collected so far are also shown in Table 2. As can be seen, the device achieve a counting error of 0.13 with is acceptable from the business point of view. These are still preliminary results but confirm that our system is able to meet its goal in a real environment. At the moment, further tests are being performed. Our goal is to determine the most usual sources of error in order to apply appropriate image processing to compensate them.

## 4. Conclusions

The industrial process of identifying and count garments in a textile warehouse (for both trolley and continuous chain systems) has been described. In order to improve the overall process, a novel system for garment identification and automatic counting has been proposed and described. This system is based on image processing to identify the hooks of the garment hangers. Unlike other artificial vision systems, in our proposal the image is generated by a laser beam. In particular, the core of our system is a device that generates an image of the hooks of the hangers from an array of sensors based on a set of phototransistors and a linear laser beam.

The result is a low-cost device (compared to traditional camera-based artificial vision systems). In addition, it can be placed at any control points of a textile warehouse since it has small size. Moreover, it works for both trolley and continuous chain mode, where the batch length is variable.

The system has been tested in real production environments. Results show that the average error is only 0.13%, *i.e.*, in average there are 13 mistakes *per* ten thousand clothing units, which is acceptable from a business point of view. Besides, it has to be taken into account that our system can not be affected by distractions, fatigue, *etc.* and can work 24/7.

Furthermore, the system performs the whole process (scanning, counting, sending information) while the trolleys are moving at their regular speed (which can be more than 1 m/s). That is, there is no need to stop or to slow down the chain (to count a trolley (or a batch in continuous chain mode), which means that the proposed system reduces the time needed in processes such as unloading trucks and batch arrangement because it is not necessary to spend time counting the number of cloths in a bath, as well as, the operating time in control points.

## Figures and Tables

**Table 1.** Real test performed in continuous chain warehouse. Results from selected days.

**Date**	**Number of cloth blocks (batch)**	**Real Number (R) of hangers (worker)**	**Estimated number from Garment counter**	**Difference (D)**	**Error (D/R%)**

10-Feb	814	50,659	50,749	90	0.18
16-Feb	663	39,573	39,641	68	0.17
02-Mar	602	35,796	35,532	264	0.83
13-Mar	1,695	91,876	91,756	120	0.13
25-Mar	1,781	115,183	114,953	230	0.20
05-Apr	1,241	85,074	84,777	297	0.35
10-Apr	1,537	97,245	97,233	12	0.01
15-Apr	1,247	83,011	82,901	110	0.13

**Table 2.** Resume of performance evaluation during 41 days (from 10 February to 15 April).

**Total number of cloth blocks (batch)**	**Real Number (R) of hangers (worker)**	**Estimated number from Garment counter**	**Difference (D)**	**Error (D/R%)**
31,417	1,940,558	1,938,023	2,535	0.13
